# BAG3 elevation inhibits cell proliferation via direct interaction with G6PD in hepatocellular carcinomas

**DOI:** 10.18632/oncotarget.6396

**Published:** 2015-11-26

**Authors:** De-Hui Kong, Si Li, Zhen-Xian Du, Chuan Liu, Bao-Qin Liu, Chao Li, Zhi-Hong Zong, Hua-Qin Wang

**Affiliations:** ^1^ Department of Biochemistry and Molecular Biology, China Medical University, Shenyang, China; ^2^ Key Laboratory of Cell Biology, Ministry of Public Health, and Key Laboratory of Medical Cell Biology, Ministry of Education, China Medical University, Shenyang, China; ^3^ Department of Endocrinology and Metabolism, The 1st Affiliated Hospital, China Medical University, Shenyang, China; ^4^ Department of Gynecology and Obstetrics, Shengjing Hospital, China Medical University, Shenyang, China

**Keywords:** BAG3, HCCs, G6PD, pentose phosphate pathway

## Abstract

Bcl-2 associated athanogene 3 (BAG3) contains multiple protein-binding motifs to mediate potential interactions with chaperons and/or other proteins, which is possibly ascribed to the multifaceted functions assigned to BAG3. The current study demonstrated that BAG3 directly interacted with glucose 6 phosphate dehydrogenase (G6PD), the rate-limiting enzyme of the pentose phosphate pathway (PPP). BAG3 suppressed the PPP flux, *de novo* DNA synthesis and cell growth in hepatocellular carcinomas (HCCs). The growth defect of HCCs with forced BAG3 expression can be rescued by enforced G6PD expression. However, BAG3 elevation did not cause a reduction in cellular NADPH concentrations, another main product of G6PD. In addition, supplement of nucleosides alone was sufficient to recover the growth defect mediated by BAG3 elevation. Collectively, the current study established a tumor suppressor-like function of BAG3 via direct interaction with G6PD in HCCs at the cellular level.

## INTRODUCTION

Bcl-2 associated athanogene (BAG) proteins share evolutionarily conserved BAG domain at their C-terminus, through which BAG proteins interact with ATPase domain of Hsc/Hsp70 family [38]. Beside conserved BAG domain, BAG3 contains multiple protein binding motifs to mediate potential interactions with chaperons and/or other proteins: a WW domain at the N-terminus, a proline-rich region (PxxP) in the central region, two IPV motifs (Ile-Pro-Val) between the WW domain and the PxxP region [10]. Normal cells except for myocytes seldom express BAG3, while its expression can be induced in many cell types in response to cell stress [11, 30, 42]. BAG3 induction is believed to serve as a protective mechanism upon cellular stress [6, 18, 25, 36–38, 42]. In addition, BAG3 expression is frequently increased in human tumors [4, 5, 8, 9, 20, 27, 28, 33]. BAG3 protein appears to exert pro-survival and anti-apoptotic roles, as its reduction promotes apoptosis of various cancer cells [4, 22–24, 27–29, 31, 36, 42, 43].

Studies on BAG3 has assigned it a variety of functions, such as regulation of cell cycle, cell survival and apoptosis, viral replication, and autophagy [2, 21, 32, 34, 39]. The modular structure with multidomain is believed to be responsible for the pleiotropic functions attributed to BAG3 [32]. We therefore explore to globally seek for novel interacting partners of BAG3 and identify direct interaction of BAG3 with Glucose-6-phosphate dehydrogenase (G6PD). G6PD is the first and rate-limiting enzyme of the pentose phosphate pathway (PPP), which is an offshoot of glucose metabolic pathway important for both glucose catabolism and macromolecular biosynthesis.

The PPP is also called the hexose monophosphate shunt, which has a crucial role in generation of ribose-5-phosphate (R5P) and nicotinamide adenine dinucleotide phosphate (NADPH). R5P is an essential precursor for *de novo* biosynthesis of nucleotides. NADPH provides the reducing equivalents for reductive biosynthesis (such as deoxyriboses and fatty acids) and is required for antioxidant defense by controlling the concentration of reduced glutathione (GSH) [35]. Cancer cells create a metabolic phenotype that is essential for quick proliferation and survival, through substantial alterations and adaptations in several energy metabolism pathways, including glucose transport, oxidative phosphorylation and the PPP [14].

The current study demonstrates that BAG3 directly interacts with G6PD, and BAG3 elevation suppresses the PPP flux and proliferation of HCCs via suppression of G6PD.

## RESULTS

### BAG3 directly interacts with G6PD in HCCs

**Figure 1:**
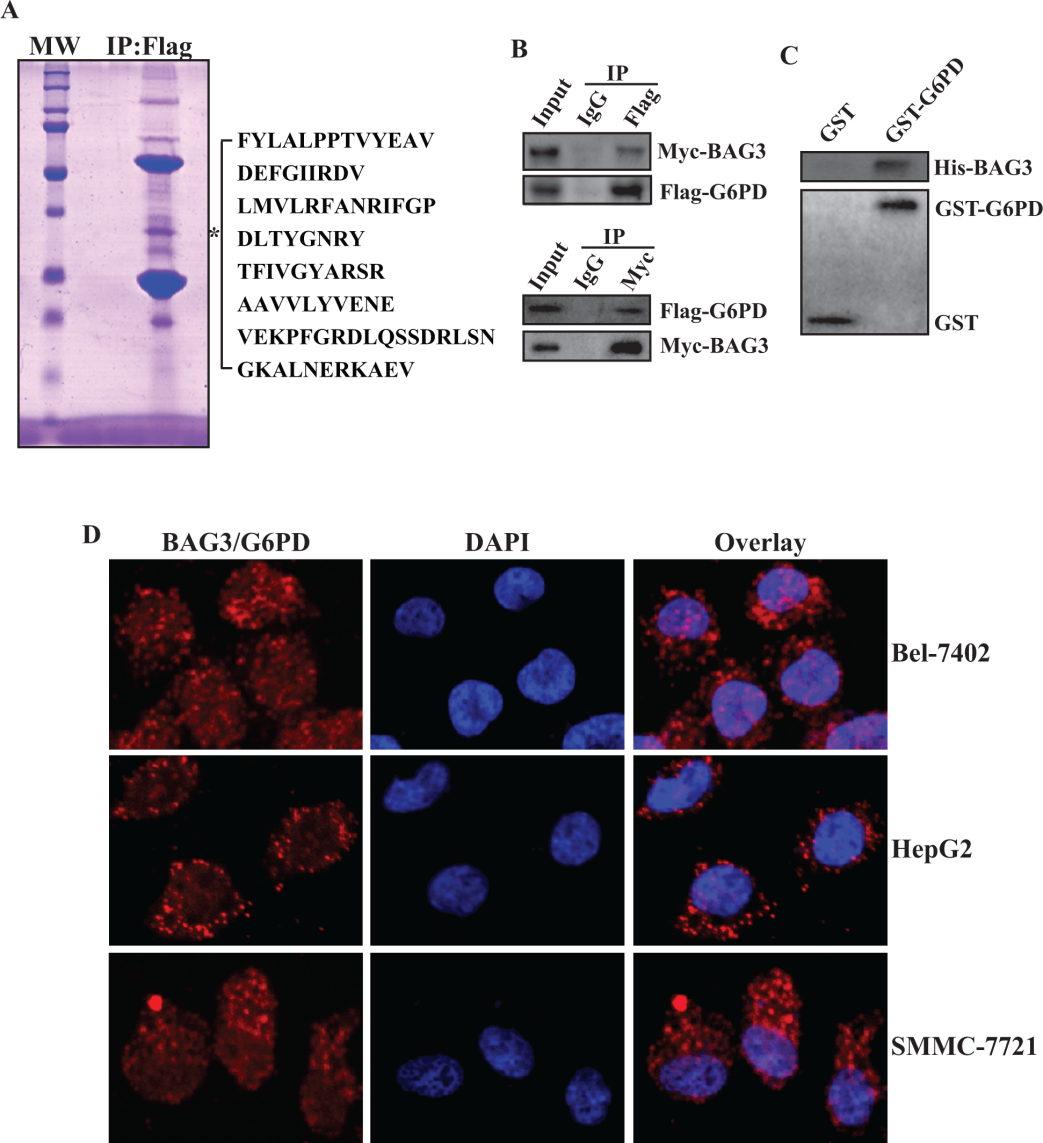
BAG3 directly interacts with G6PD (**A**) Total proteins were isolated from HEK293 cells transfected with Flag-tagged BAG3 construct. BAG3-containing complexes were immunoprecipitated by an anti-Flag antibody and separated by SDS-PAGE. Gel was stained with Coomassie blue and protein bands were analyzed using peptide mass fingerprinting. The star denotes a prominent polypeptide of about 60-KDa and polypeptides of G6PD identified by mass spectrometry are summarized. (**B**) HEK293 cells were co-transfected with Myc-tagged BAG3 and Flag-tagged G6PD constructs. Reciprocal co-immunoprecipitation of BAG3 and G6PD was performed. (**C**) Purified His-BAG3 recombinant proteins were incubated with GST-G6PD or GST immobilized on beads. Beads-bound (pulldown) proteins were analyzed by western blot. (**D**) Endogenous direct interaction of BAG3 and G6PD in HCCs was analyzed using Duolink PLA, and representative images were provided.

Global screen for interactive partners of BAG3 revealed an apparent band with about 60-kDa molecular mass in BAG3 containing complexes [17] (Figure 1A). Subsequent peptide mass fingerprinting identified one of the polypeptides as G6PD, based on 8 peptides with sequence coverage of 17.7% (Figure 1A). To verify the interaction between BAG3 and G6PD, HEK293 cells were co-transfected with BAG3 and G6PD expressing vectors. Reciprocal immunoprecipitation confirmed that BAG3 formed complexes with G6PD (Figure 1B). Pull-down assays demonstrated the interaction between purified GST-G6PD and His-BAG3 recombinant proteins, but not GST and His-BAG3 (Figure 1C). In addition, *in situ* proximity ligation assay (PLA) demonstrated that direct interaction of endogenous BAG3 and G6PD in HCCs including Bel-7402, HepG2 and SMMC-7721 cells (Figure 1D).

### BAG3 elevation inhibits dimerization and activity of G6PD in HCCs

**Figure 2:**
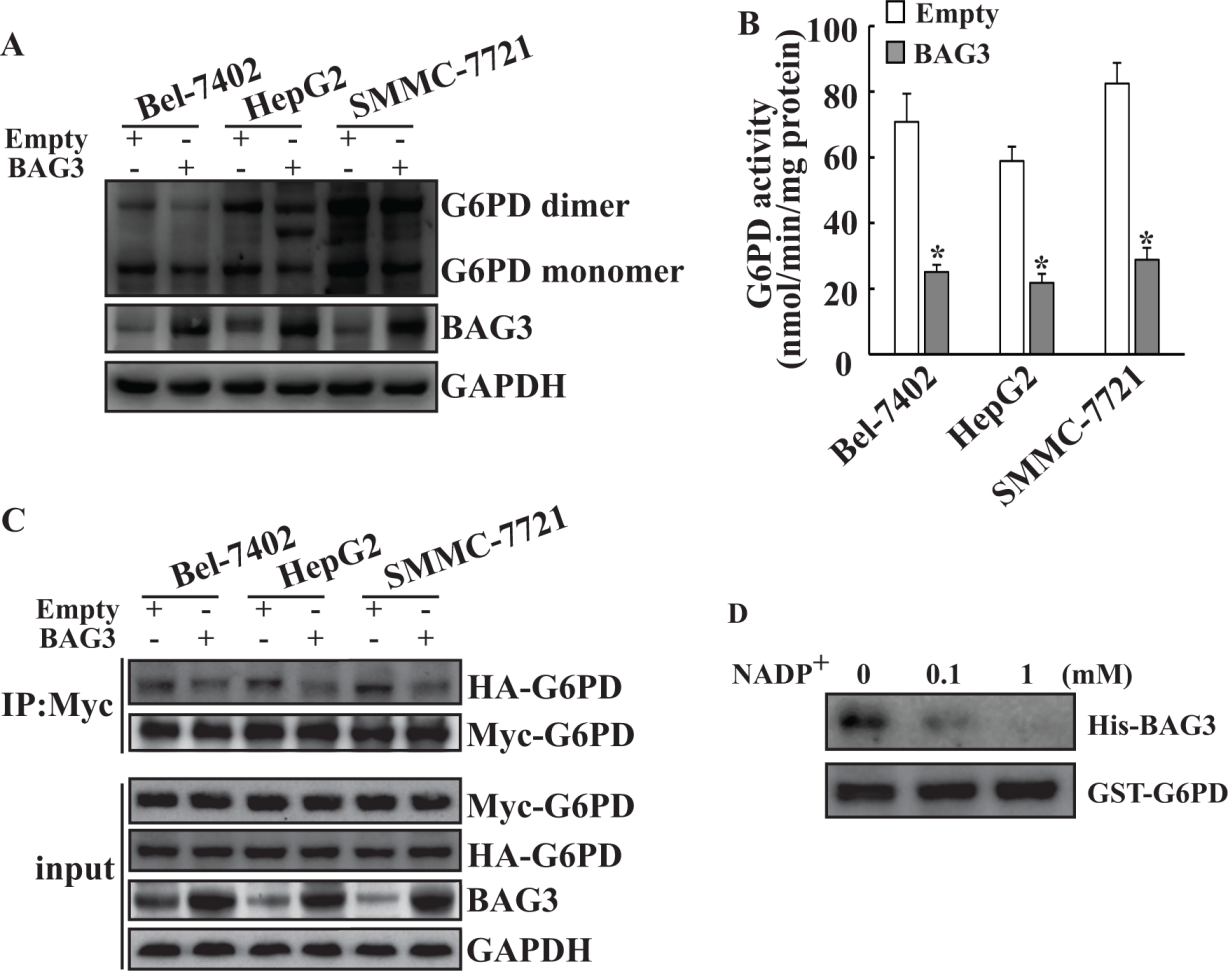
Forced BAG3 expression suppresses dimerization and activity of G6PD in HCCs (**A**) HCCs cells were infected with recombinant retrovirus containing empty or BAG3 construct. Total proteins were isolated and separated by native-PAGE. (**B**) Total proteins were isolated from HCCs transduced with empty or BAG3 constructs. Cellular G6PD activity was measured and normalized by cellular proteins. (**C**) Empty or BAG3-transduced HCCs were co-transfected with HA-tagged and Myc-tagged G6PD constructs. Total proteins were isolated and co-immunoprecipitation was performed using anti-Myc antibody. (**D**) Purified His-BAG3 recombinant proteins were incubated with GST-G6PD or GST immobilized on beads in the presence of increasing amounts of NADP^+^ (0, 0.1 and 1 mM). Beads-bound (pulldown) proteins were analyzed by Western blot.

To investigate the influence of BAG3 on G6PD, Bel-7402, HepG2 and SMMC-7721 cells were transduced with the *BAG3* gene using retroviral vectors. Western blot analyses found that BAG3 elevation decreased both dimer and monomer of G6PD levels in HCCs (Figure 2A). G6PD activity assays demonstrated that G6PD activities were decreased in HCCs with forced BAG3 expression (Figure 2B). To investigate the influence of BAG3 on G6PD dimer formation, HCCs were co-transfected with G6PD constructs with HA and Myc epitope tags. Immunoprecipitation demonstrated that interaction between HA-G6PD and Myc-G6PD was significantly decreased in HCCs with forced BAG3 expression (Figure 2C). Nicotinamide adenine dinucleotide phosphate (NADP^+^) functions as the cofactor for the formation of G6PD holoenzyme [15]. GST pull-down assays demonstrated that the interaction between BAG3 and G6PD was suppressed by NADP^+^ in a dose-dependent manner (Figure 2D).

### BAG3 elevation suppresses DNA biosynthesis without alteration of cellular NADPH levels in HCCs

**Figure 3:**
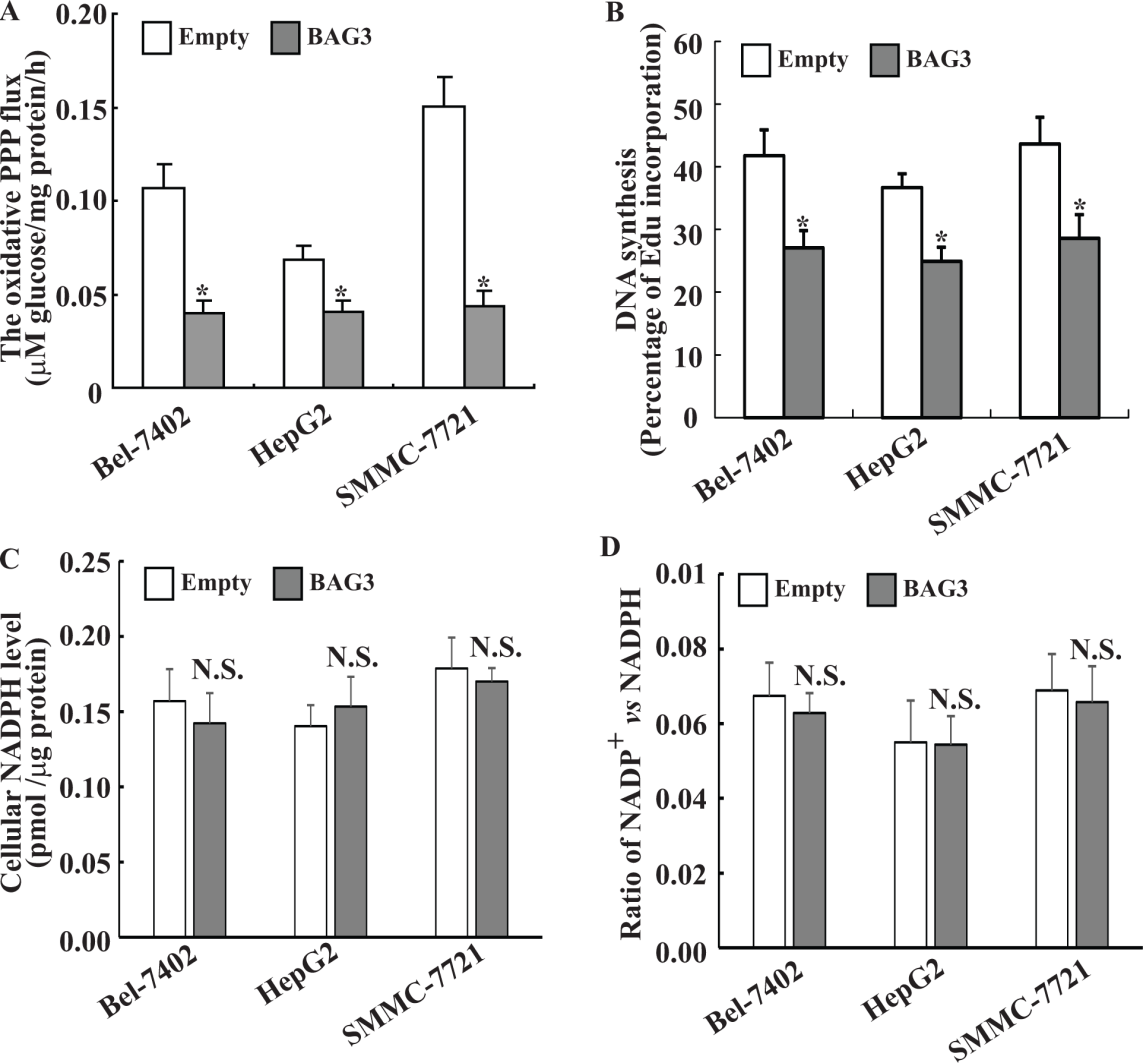
Forced BAG3 expression suppressed the PPP in HCCs (**A**) Empty or BAG3-transdued HCCs were cultured in medium containing [2–^13^C] glucose, and the PPP flux was analyzed using NMR. (**B**) HCCs were transduced with retrovirus containing empty or BAG3 construct for 48 h. *de novo* DNA synthesis was analyzed using EdU incorporation. (**C–D**) HCCs were transduced with retrovirus containing empty or BAG3 construct for 48 h. Cellular NADPH (C) levels and NADPH/NADP^+^ ratio (D) were measured and normalized by cellular proteins.

As G6PD is the pacesetter of the PPP, we assessed whether BAG3 might have any influence on the glucose flux through this pathway. The PPP flux was significantly slowed down in HCCs with forced BAG3 expression when compared with their control partners (Figure 3A). As the PPP generates NADPH and R5P, both of which are important precursors for DNA biosynthesis, *de novo* biosynthesis of DNA was then investigated using Edu incorporation. EdU incorporation rate was significantly decreased in HCCs with forced BAG3 expression when compared with their control partners (Figure 3B). Unexpectedly, no obvious alterations of cellular NADPH (Figure 3C), as well as NADP^+^/NADPH ratio (Figure 3D) were observed in HCCs with forced BAG3 expression.

### BAG3 elevation results in growth inhibition of HCCs

**Figure 4:**
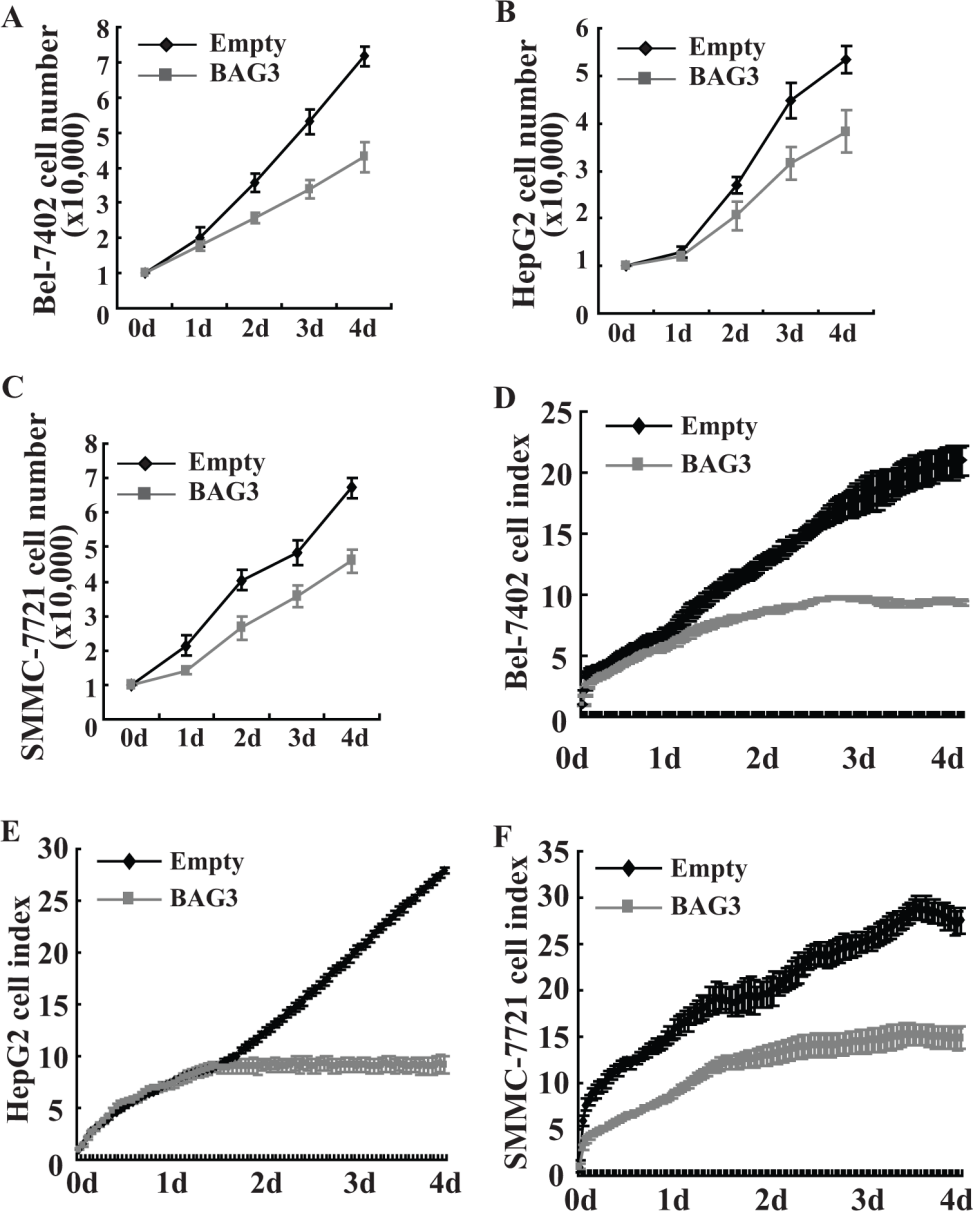
Forced BAG3 expression suppresses the proliferation of HCCs (**A–C**) Bel-7402 (A), HepG2 (B) and SMMC-7721 (C) cells transduced with retrovirus containing empty or BAG3 construct were plated on 6-well plate, and cell numbers were counted daily for 4 days. (**D–F**) Bel-7402 (D), HepG2 (E) and SMMC-7721 (F) cells transduced with retrovirus containing empty or BAG3 construct were plated on E-plate, and real-time cell indexes were analyzed using RTCA.

Cell count revealed that proliferation of HCCs with forced BAG3 expression was slowed down when compared with their control partners (Figure 4A–4C). Cell proliferation was also analyzed using RTCA, and reduction of cell indexes was observed in Bel-7402 (Figure 4D), HepG2 (Figure 4E) and SMMC-7721 (Figure 4F) with forced BAG3 expression. TUNEL staining manifested the absence of detectable apoptosis in HCCs with forced BAG3 expression (data not shown).

### BAG3 regulates DNA biosynthesis and cell growth via G6PD in HCCs

**Figure 5:**
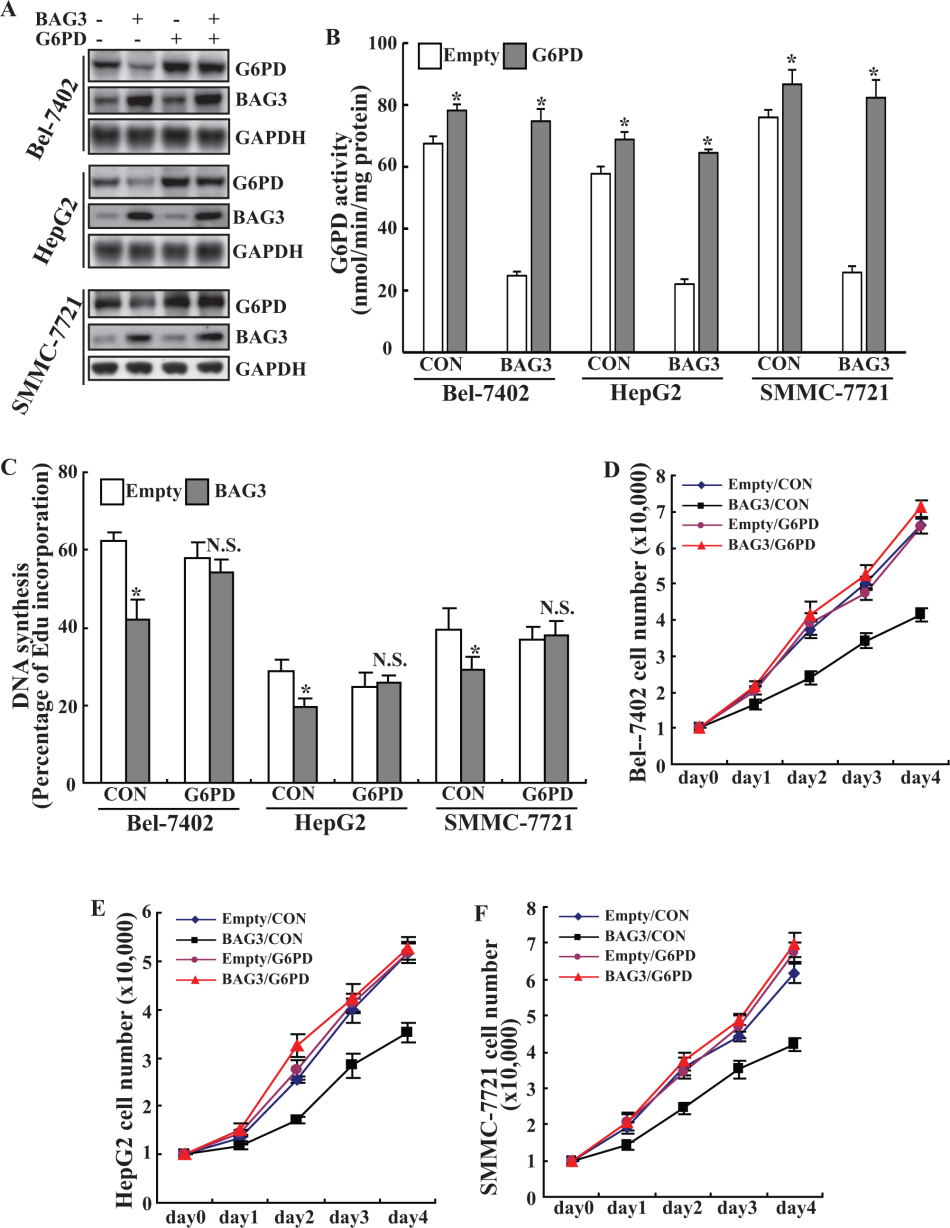
G6PD rescues the growth defect of HCCs mediated by BAG3 elevation (**A**) Empty or BAG3-transduced HCCs were infected with retrovirus containing G6PD construct and Western blot analysis was performed using the indicated antibodies. (**B**) Empty or BAG3-transduced HCCs were infected with retrovirus containing G6PD construct and G6PD activities were analyzed. (**C**) Empty or BAG3-transduced HCCs were infected with retrovirus containing G6PD construct and *de novo* DNA synthesis was analyzed using Edu incorporation. (**D**–**F**) Empty or BAG3-transduced Bel-7402 (D), HepG2 (E) and SMMC-7721 (F) cells were infected with retrovirus containing G6PD construct and cell numbers were counted daily.

To ascertain that the growth retardation is due to G6PD, G6PD expression (Figure 5A) and activity (Figure 5B) was restored by retrovirus-mediated gene transfer (Figure 5A). Forced expression of G6PD almost completely restored DNA synthesis in HCCs with forced BAG3 expression, while had a minimal effect on their control partners (Figure 5C). Cell counts demonstrated that forced G6PD expression completely recovered the growth inhibition mediated by BAG3 overexpression in Bel-7402 (Figure 5D), HepG2 (Figure 5E) and SMMC-7721 (Figure 5F).

### Supplement of nucleosides rescues growth inhibition of HCCs mediated by BAG3 overexpression

**Figure 6:**
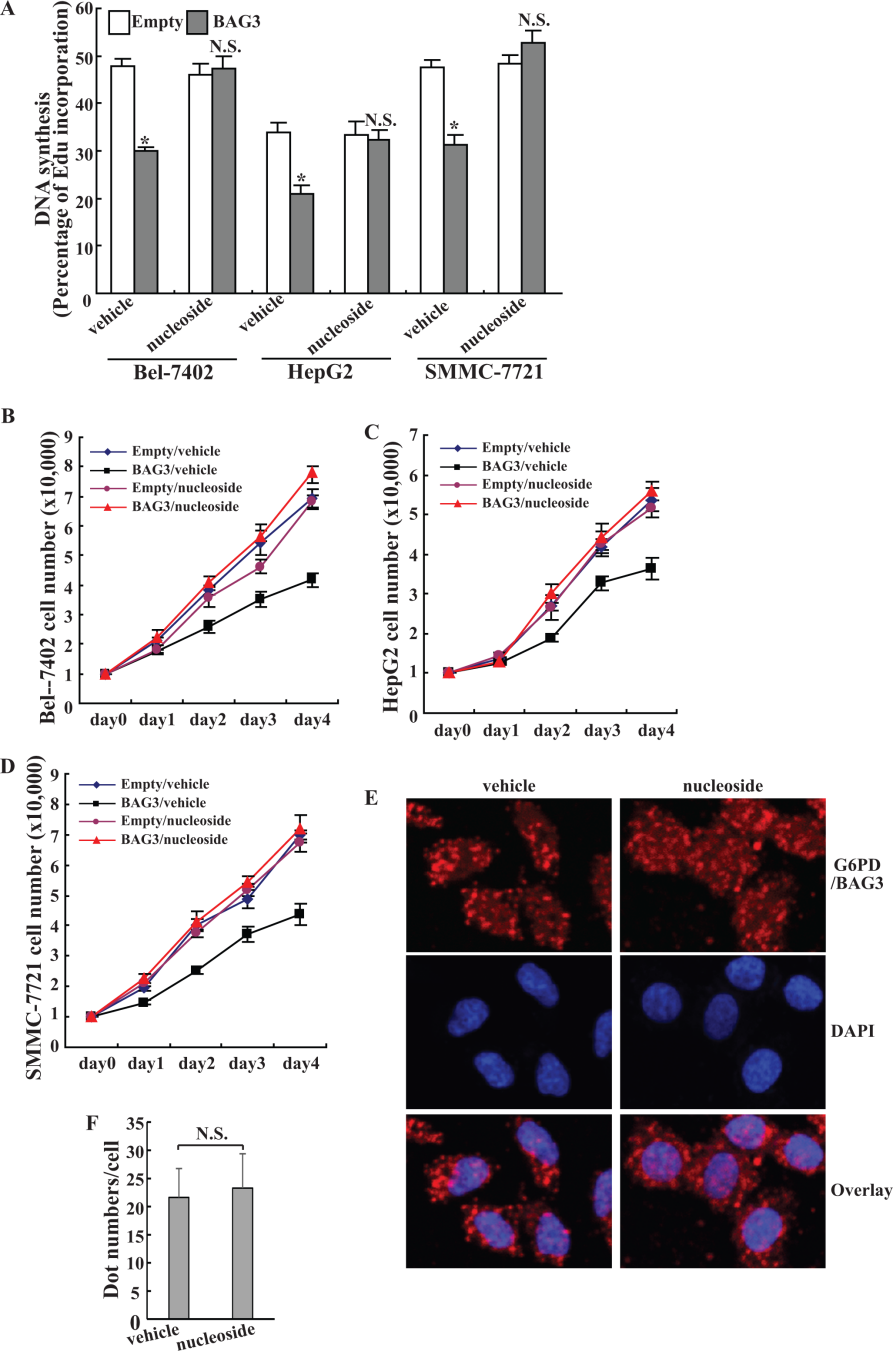
Nucleosides are sufficient to rescue the growth defect of HCCs mediated by BAG3 elevation (**A**) Nascent DNA in empty or BAG3-transduced HCCs was labeled using EdU for 5 h in the absence or presence of 10 μM uridine and 10 μM hypoxathine. *de novo* DNA synthesis was analyzed by Edu incorporation rate. (**B–D**) Empty or BAG3-transduced Bel-7402 (B), HepG2 (C) and SMMC-7721 (**D**) cells were cultured in the absence or presence of 10 μM uridine and 10 μM hypoxathine. Cell numbers were counted daily. (**E**) Bel-7402 cells were cultured in the absence or presence of 10 μM uridine and 10 μM hypoxathine for 8 h. Interaction of BAG3 and G6PD was analyzed using DuoLink PLA, and representative images were provided. (**F**) DuoLink PLA dots (E) in Bel-7402 cells cultured in the absence or presence of 10 μM uridine and 10 μM hypoxathine were counted. Results shown represent mean ± SD from at least 50 cells/group.

Cells can uptake nucleosides from culture medium and convert them to the corresponding nucleotides, thus bypassing *de novo* synthesis of ribose for cell proliferation [7]. Replenishment of nucleoside significantly increased DNA synthesis in HCCs with forced BAG3 expression (Figure 6A). In the presence of nucleosides, no obvious differences in DNA synthesis were observed in HCCs transferred with empty vector and BAG3 (Figure 6A). Cell count demonstrated that nucleosides completely restored cell proliferation in Bel-7402 (Figure 6B), HepG2 (Figure 6C) and SMMC-7721 (Figure 6D) with forced BAG3 expression. Duolink PLA demonstrated that the interaction between BAG3 and G6PD was unaltered in the presence of nucleosides in Bel-7402 cells (Figure 6E–6F).

## DISCUSSION

Redistribution of glucose utilization is one key characteristic of cancer cells to coordinate nutrient utilization with cell physiology [41]. One of mechanisms used by cells to support the rapid proliferation is redirection of glucose toward the PPP, which may protect cells from reactive oxygen species (ROS) by generation of the reduced form of glutathione using NADPH [3]. The first rate-limiting enzyme of the PPP is G6PD, which is highly regulated by many signaling pathways at multiple levels, including transcription, translation, post-translation, intracellular location, and interactions with other proteins [35]. The current study demonstrated that BAG3 directly interacted with G6PD (Figure 1) and suppressed G6PD activity in HCCs (Figure 2B). A band between the G6PD dimer and monomer was obviously detected in HepG2 and SMMC-7721 cells, but undetectable in Bel-7402 cells (Figure 2A). Therefore, posttranslational modifications of G6PD such as glycosylation might take place in a cell specific manner in HCCs. A dimer or tetramer but not a monomer form of G6PD protein in mammalian cells is active [1, 16]. Consistent with these reports, we found that BAG3 hindered dimerization of G6PD in HCCs (Figure 2C). NADP^+^ binds to G6PD and functions as a cofactor for the formation of G6PD holoenzyme [15]. NADP^+^ suppressed the interaction of BAG3 and G6PD in a dose-dependent pattern (Figure 2D), suggesting that NADP^+^ and BAG3 might competitively bind to G6PD. Further screening the interactive domains of G6PD and BAG3 might clarify whether BAG3 prohibits the formation of G6PD holoenzyme via competing with NADP^+^ for binding to G6PD.

The major products of the PPP are R5P and NADPH, both of which are precursors for DNA biosynthesis. Consistent with suppression of the PPP flux (Figure 3A), BAG3 prohibited DNA biosynthesis (Figure 3B) and cell proliferation (Figure 4) in HCCs. Forced G6PD overexpression completely recovered DNA synthesis (Figure 5C) and growth defect (Figure 5D–5F) mediated by BAG3 elevation, indicating that BAG3 elevation might suppress the PPP flux and proliferation of HCCs via direct interaction with G6PD in HCCs. G6PD overexpression had no obvious effects on DNA biosynthesis (Figure 5C) and proliferation (Figure 5D–5F) of control HCCs. Du *et al.* also demonstrated that proliferation of U2OS cells was not affected by G6PD overexpression [7]. These data were unexpected as G6PD functions as oncogenic roles in tumor cells including HCCs [35]. The PPP shunts G6P from ATP generation to macromolecular biosynthesis, which is hyperactive in cancer cells either directly or indirectly [40]. The balance between macromolecule biosynthesis and ATP generation are critical for cell proliferation. Therefore, it is possible that G6PD activity in control HCCs is enough to catalyze generation of R5P and NADPH using G6P as substrate, in case that glucose uptake is not increased.

Consistent with control U2OS and MEF cells [7], the current study demonstrated that addition of nucleosides alone had no effect on proliferation of control HCCs (Figure 6B–6D). BAG3 did not decreased intracellular NADPH (Figure 3C) and NADP^+^/NADPH ratio (Figure 3D). Although the PPP flux is the major source for NADPH generation in animals, there are several other processes involved in generation of intracellular NADPH [13]. Beside glucose catabolism via the PPP, glutaminolysis is another major mechanism for NADPH production in tumor cells [44]. Further investigation of glutamine metabolism might clarify whether BAG3 affects NADPH generation via glutaminolysis. Addition of nucleosides alone was sufficient to restore *de novo* DNA biosynthesis (Figure 6A) and growth of HCCs with forced BAG3 expression (Figure 6B–6D), while had no obvious influence on binding of BAG3 to G6PD (Figure 6E–6F). Therefore, lack of R5P might be the critical missing factor required for cell growth in HCCs with forced BAG3 expression. Suppression of DNA synthesis (Figure 3A) was less intensive than decreases in G6PD activity (Figure 2B), indicating that an alternative route bypass G6PD might be present to generate R5P in HCCs with forced BAG3 expression. Consistent with our data, G6PD activity is reported to be dispensable for pentose synthesis in mouse embryo stem cells [26].

The current study demonstrated that BAG3 elevation exhibited a tumor suppressor-like effect on proliferation of HCCs at the cellular level. The inhibitory role of BAG3 elevation in cell proliferation at the cellular level raises the question of why BAG3 is highly expressed in HCCs [45]. Similar phenomena were also observed in glioblastoma. BAG3 expression was increased in glioblastoma tissues [8], while overexpression of BAG3 resulted in a remarkable decrease in the colony formation, a tumor suppressor-like effect at the cellular level in glioblastoma [12]. BAG3 contains a modular structure, through which BAG3 may interact with distinct partners. It is reasonable that the extent of BAG3 expression (overexpression or knockdown) may alter its binding partner(s) differently. Therefore, the specificity of BAG3 interacting clients could justify the difference in the phenotypic outcome of different biological behaviors. Supplement of nucleosides was enough to recover proliferation of HCCs with BAG3 elevation (Figure 6B–6D). HCCs might have sufficient nucleoside supply and thereby bypass the requirement of *de novo* nucleoside biosynthesis during tumor formation *in vivo*. Alternatively, the beneficial influences associated with BAG3 elevation upon stressful stimuli may outweigh any proliferative defects under normal condition. As a fact, we previously reported that BAG3 promoted survival of HepG2 cells upon exposure to proteasome inhibitors via activation of autophagy [21].

In summary, our data identified that BAG3 suppressed the PPP flux via direct interaction with G6PD, by which BAG3 elevation suppressed proliferation of HCCs at the cellular level.

## MATERIALS AND METHODS

### Culture of cell lines

HEK293, Bel-7402, HepG2 and SMMC-7721 cell lines were maintained in DMEM (Sigma-Aldrich, Saint Louis, MO) and supplemented with 10% fetal bovine serum (FBS, ExCell Biology Inc., Shanghai, China).

### Gene expression transferred by recombinant retrovirus infection

To study the function of BAG3 and G6PD, retroviral vectors were constructed (Shanghai GeneChem Co., Ltd., Shanghai, China). GFP or RFP-retroviral vectors were used as a negative control. All retroviral vectors expressed GFP or RFP, which allowed us to measure the infection efficiency. HCCs were infected at 20 m.o.i with recombinant virus expressing BAG3 or G6PD for 48 h. Then cells were harvested and subjected to further analysis.

### Coimmunoprecipitation assay and peptide mass fingerprinting

Coimmunoprecipitation assay was performed as previously reported [17]. Coomassie-stained protein bands were excised and analyzed by Matrix-assisted laser desorption ionization time-of-flight (MALDI-TOF) mass spectrometry as previously described [17].

### Western blot analysis and immunoprecipitation

Cells were lysed in lysis buffer containing 20 mM Tris-HCl, 150 mM NaCl, 2 mM EDTA, 1% Triton-X100 and protease inhibitor cocktail (Sigma-Aldrich, Saint Louis, MO). Extracted protein amounts were quantified using the BCA protein assay kit (Pierce). Equivalent amounts of protein (25 μg) were separated using 12% SDS-PAGE and transferred to PVDF membrane (Millipore Corporation, Billerica, MA). For immunoprecipitation, cell lysates were pre-cleared with protein A-Sepharose CL-4B (Amersham Biosciences, Uppsala, Sweden), then incubated overnight at 4°C with Myc or Flag antibody and protein A-Sepharose. The immunoprecipitates were washed three times with lysis buffer and analyzed by Western blot analysis, which was performed using primary antibodies against G6PD (Abcam, Cambridge, UK), BAG3 (Abcam, Cambridge, UK) or GAPDH (Chemicon, Bedford, MA), horseradish peroxidase (HRP)-conjugated anti-mouse or anti-rabbit secondary antibodies (Amersham Biosciences, UK) and ECL solutions (Amersham Biosciences, UK).

### *In situ* Proximity ligation assay (PLA)

*In situ* proximity ligation assay (PLA) was performed using Duolink PLA (Sigma-Aldrich, Saint Louis, MO) according to the manufacturer's protocol. PLA probes were diluted in 0.1% Triton X-100/PBS/1% fetal calf serum and incubated in a pre-heated humidity chamber for 1 h at 37°C, followed by hybridization, ligation, amplification, and detection. Fluorescence was visualized under a confocal microscopy (TCS SP5 Leica Microsystems).

### Cell proliferation assay

Cells were seeded into 6-well plates at a density of 10,000 cells per well in 2 ml of medium supplemented with 10% FBS. The medium was changed every 2 days. Cell number at the indicated time points was determined by counting using a haemocytometer.

### Growth curve assays using real-time cell analyzer (RTCA)

To analyze the cells continuously over time, growth curve assays were performed in real time in quadruplicate with the xCELLigence system (ACEA Bioscience, San Diego, CA) as previously described [19]. Briefly, 5,000 cells per well were seeded in RTCA E-plates (ACEA Bioscience, San Diego, CA), the electrical impedance in each well was measured continuously. The shift of the electrical impedance is expressed as the cell index, which is a parameter of cell viability.

### *De novo* DNA biosynthesis

DNA synthesis was determined by Click-iT Edu Assay Kit (Invitrogen, CA) according to the manufacturer's protocol. Briefly, HCCs were incubated with 10 μM of nucleoside analog 5-ethynyl-2′ deoxyuridine (Edu) in complete medium for 4 hours. After washing and fixation, incorporated Edu was labeled with Alexa Fluor 555 azide in the provided reaction buffer for 30 min.

### Measurement of the PPP flux

HCCs were cultured in medium containing [2–^13^C] glucose (Sigma-Aldrich), and the PPP flux was measured on the basis of the rate of glucose consumption and ^13^C labeling patterns in lactate (incorporated into carbon 2 and carbon 3 generated by glycolysis and the PPP, respectively) determined by nuclear magnetic resonance (NMR) spectroscopy [15].

### Cellular NADPH measurement

Cellular NADPH concentrations were determined by a NADPH assay kit (Abcam, Cambridge, UK) according to the manufacturer's protocol. Briefly, HCCs were lysed in 400 μl extraction buffer provided and half of this lysate were heated at 60°C for 30 min to decompose NADP^+^. Under these conditions NADPH remained intact, according to the manufacturer. A portion of this heated-extract was used to determine NADPH concentration. Another portion was used in protein concentration determination for normalization.

### G6PD enzyme activity

G6PD enzyme activity was determined used G6PD activity assay kit (Cayman Chemical, MI) according to the manufacturer's instructions. G6PDH catalyzes the oxidation of glucose-6-phosphate to 6-phospho-D-gluconate, along with the concomitant reduction of NADP^+^ to NADPH. NADPH reacts with the fluorometric detector to yield a highly fluorescent product which can be analyzed with an excitation wavelength of 530 nm and an emission wavelength of 595 nm. Enzyme activities were normalized on the basis of protein concentration, which was determined by a BCA protein assay kit (Pierce).

### Supplement of nucleosides

The current study used combination of uridine and hypoxathine (Sigma-Aldrich) for nucleosides supplement. Uridine and hypoxathine are the pyrimidine base for UMP and purine base for IMP generation, respectively. UMP and IMP are the firstly fully formed pyrimidine and purine nucleotide *in vivo*, from which all nucleotides used for DNA and RNA synthesis can be produced.

### Statistics

The statistical significance of the difference was analyzed by ANOVA and post hoc Dunnett's test. Statistical significance was defined as *P* < 0.05. All experiments were repeated three times, and data were expressed as the mean ± SD (standard deviation) from a representative experiment.

## References

[1] Au SW, Gover S, Lam VM, Adams MJ (2000). Human glucose-6-phosphate dehydrogenase: the crystal structure reveals a structural NADP (+) molecule and provides insights into enzyme deficiency. Structure.

[2] Behl C (2011). BAG3 and friends: co-chaperones in selective autophagy during aging and disease. Autophagy.

[3] Bensaad K, Tsuruta A, Selak MA, Vidal MN, Nakano K, Bartrons R, Gottlieb E, Vousden KH (2006). TIGAR, a p53-inducible regulator of glycolysis and apoptosis. Cell.

[4] Bonelli P, Petrella A, Rosati A, Romano MF, Lerose R, Pagliuca MG (2004). BAG3 protein regulates stress-induced apoptosis in normal and neoplastic leukocytes. Leukemia.

[5] Chiappetta G, Ammirante M, Basile A, Rosati A, Festa M, Monaco M, Vuttariello E, Pasquinelli R, Arra C, Zerilli M, Torado M, Stassi G, Pezzullo L (2007). The Antiapoptotic Protein BAG3 Is Expressed in Thyroid Carcinomas and Modulates Apoptosis Mediated by Tumor Necrosis Factor-Related Apoptosis-Inducing Ligand. J Clin Endocrinol Metab.

[6] Doong H, Vrailas A, Kohn EC (2002). What's in the ‘BAG’?—A functional domain analysis of the BAG-family proteins. Cancer Lett.

[7] Du W, Jiang P, Mancuso A, Stonestrom A, Brewer MD, Minn AJ, Mak TW, Wu M, Yang X (2013). TAp73 enhances the pentose phosphate pathway and supports cell proliferation. Nat Cell Biol.

[8] Festa M, Del Valle L, Khalili K, Franco R, Scognamiglio G, Graziano V, De Laurenzi V, Turco MC, Rosati A (2011). BAG3 protein is overexpressed in human glioblastoma and is a potential target for therapy. Am J Pathol.

[9] Franco R, Scognamiglio G, Salerno V, Sebastiani A, Cennamo G, Ascierto PA, Botti G, Turco MC, Rosati A (2012). Expression of the anti-apoptotic protein BAG3 in human melanomas. J Invest Dermatol.

[10] Gamerdinger M, Carra S, Behl C (2011). Emerging roles of molecular chaperones and co-chaperones in selective autophagy: focus on BAG proteins. J Mol Med (Berl).

[11] Gentilella A, Passiatore G, Deshmane S, Turco MC, Khalili K (2008). Activation of BAG3 by Egr-1 in response to FGF-2 in neuroblastoma cells. Oncogene.

[12] Gentilella A, Khalili K (2011). BAG3 expression in glioblastoma cells promotes accumulation of ubiquitinated clients in an Hsp70-dependent manner. J Biol Chem.

[13] Hanukoglu I, Rapoport R (1995) (2011). Routes and regulation of NADPH production in steroidogenic mitochondria. Endocr Res.

[14] Hitosugi T, Zhou L, Elf S, Fan J, Kang HB, Seo JH, Shan C, Dai Q, Zhang L, Xie J, Gu TL, Jin P, Alečković M (2012). Phosphoglycerate mutase 1 coordinates glycolysis and biosynthesis to promote tumor growth. Cancer Cell.

[15] Jiang P, Du W, Wang X, Mancuso A, Gao X, Wu M, Yang X (2011). p53 regulates biosynthesis through direct inactivation of glucose-6-phosphate dehydrogenase. Nat Cell Biol.

[16] Kletzien RF, Harris PK, Foellmi LA (1994). Glucose-6-phosphate dehydrogenase: a “housekeeping” enzyme subject to tissue-specific regulation by hormones, nutrients, and oxidant stress. Faseb J.

[17] Kong DH, Zhang Q, Meng X, Zong ZH, Li C, Liu BQ, Guan Y, Wang HQ (2013). BAG3 sensitizes cancer cells exposed to DNA damaging agents via direct interaction with GRP78. Biochim Biophys Acta.

[18] Lee MY, Kim SY, Choi JS, Choi YS, Jeon MH, Lee JH, Kim IK, Lee JH (2002). Induction of Bis, a Bcl-2-binding protein, in reactive astrocytes of the rat hippocampus following kainic acid-induced seizure. Exp Mol Med.

[19] Li S, Zhang HY, Wang T, Meng X, Zong ZH, Kong DH, Wang HQ, Du ZX (2014). BAG3 promoted starvation-induced apoptosis of thyroid cancer cells via attenuation of autophagy. J Clin Endocrinol Metab.

[20] Liao Q, Ozawa F, Friess H, Zimmermann A, Takayama S, Reed JC, Kleeff J, Büchler MW (200). The anti-apoptotic protein BAG-3 is overexpressed in pancreatic cancer and induced by heat stress in pancreatic cancer cell lines. FEBS Lett.

[21] Liu BQ, Du ZX, Zong ZH, Li C, Li N, Zhang Q, Kong DH, Wang HQ (2013). BAG3-dependent noncanonical autophagy induced by proteasome inhibition in HepG2 cells. Autophagy.

[22] Liu P, Xu B, Li J, Lu H (2009). BAG3 gene silencing sensitizes leukemic cells to Bortezomib-induced apoptosis. FEBS Lett.

[23] Meng Q, Xia C, Fang J, Rojanasakul Y, Jiang BH (2006). Role of PI3K and AKT specific isoforms in ovarian cancer cell migration, invasion and proliferation through the p70S6K1 pathway. Cell Signal.

[24] Obata K, Morland SJ, Watson RH, Hitchcock A, Chenevix-Trench G, Thomas EJ, Campbell IG (1998). Frequent PTEN/MMAC mutations in endometrioid but not serous or mucinous epithelial ovarian tumors. Cancer Res.

[25] Pagliuca MG, Lerose R, Cigliano S, Leone A (2003). Regulation by heavy metals and temperature of the human BAG-3 gene, a modulator of Hsp70 activity. FEBS Lett.

[26] Pandolfi PP, Sonati F, Rivi R, Mason P, Grosveld F, Luzzatto L (1995). Targeted disruption of the housekeeping gene encoding glucose 6-phosphate dehydrogenase (G6PD): G6PD is dispensable for pentose synthesis but essential for defense against oxidative stress. EMBO J.

[27] Romano MF, Festa M, Pagliuca G, Lerose R, Bisogni R, Chiurazzi F, Storti G, Volpe S, Venuta S, Turco MC, Leone A (2003). BAG3 protein controls B-chronic lymphocytic leukaemia cell apoptosis. Cell Death Differ.

[28] Romano MF, Festa M, Petrella A, Rosati A, Pascale M, Bisogni R, Poggi V, Kohn EC, Venuta S, Turco MC, Leone A (2003). BAG3 protein regulates cell survival in childhood acute lymphoblastic leukemia cells. Cancer Biol Ther.

[29] Rosati A, Ammirante M, Gentilella A, Basile A, Festa M, Pascale M, Marzullo L, Belisario MA, Tosco A, Franceschelli S, Moltedo O, Pagliuca G, Lerose R (2007). Apoptosis inhibition in cancer cells: a novel molecular pathway that involves BAG3 protein. Int J Biochem Cell Biol.

[30] Rosati A, Leone A, Del Valle L, Amini S, Khalili K, Turco MC (2007). Evidence for BAG3 modulation of HIV-1 gene transcription. J Cell Physiol.

[31] Rosati A, Khalili K, Deshmane SL, Radhakrishnan S, Pascale M, Turco MC, Marzullo L (2009). BAG3 protein regulates caspase-3 activation in HIV-1-infected human primary microglial cells. J Cell Physiol.

[32] Rosati A, Graziano V, De Laurenzi V, Pascale M, Turco MC (2011). BAG3: a multifaceted protein that regulates major cell pathways. Cell Death Dis.

[33] Rosati A, Bersani S, Tavano F, Dalla Pozza E, De Marco M, Palmieri M, De Laurenzi V, Franco R, Scognamiglio G, Palaia R, Fontana A, di Sebastiano P, Donadelli M (2012). Expression of the antiapoptotic protein BAG3 is a feature of pancreatic adenocarcinoma and its overexpression is associated with poorer survival. Am J Pathol.

[34] Sato N, Tsunoda H, Nishida M, Morishita Y, Takimoto Y, Kubo T, Noguchi M (2000). Loss of heterozygosity on 10q23. 3 and mutation of the tumor suppressor gene PTEN in benign endometrial cyst of the ovary: possible sequence progression from benign endometrial cyst to endometrioid carcinoma and clear cell carcinoma of the ovary. Cancer Res.

[35] Stanton RC (2012). Glucose-6-phosphate dehydrogenase, NADPH, and cell survival. IUBMB Life.

[36] Tabuchi Y, Ando H, Takasaki I, Feril LB, Zhao QL, Ogawa R, Kudo N, Tachibana K, Kondo T (2007). Identification of genes responsive to low intensity pulsed ultrasound in a human leukemia cell line Molt-4. Cancer Lett.

[37] Takayama S, Xie Z, Reed JC (1999). An evolutionarily conserved family of Hsp70/Hsc70 molecular chaperone regulators. J Biol Chem.

[38] Takayama S, Reed JC (2001). Molecular chaperone targeting and regulation by BAG family proteins. Nat Cell Biol.

[39] Tashiro H, Blazes MS, Wu R, Cho KR, Bose S, Wang SI, Li J, Parsons R, Ellenson LH (1997). Mutations in PTEN are frequent in endometrial carcinoma but rare in other common gynecological malignancies. Cancer Res.

[40] Tong X, Zhao F, Thompson CB (2009). The molecular determinants of de novo nucleotide biosynthesis in cancer cells. Curr Opin Genet Dev.

[41] Vander Heiden MG, Cantley LC, Thompson CB (2009). Understanding the Warburg effect: the metabolic requirements of cell proliferation. Science.

[42] Wang HQ, Liu HM, Zhang HY, Guan Y, Du ZX (2008). Transcriptional upregulation of BAG3 upon proteasome inhibition. Biochem Biophys Res Commun.

[43] Wang HQ, Liu BQ, Gao YY, Meng X, Guan Y, Zhang HY, Du ZX (2009). Inhibition of the JNK signalling pathway enhances proteasome inhibitor-induced apoptosis of kidney cancer cells by suppression of BAG3 expression. Br J Pharmacol.

[44] Wise DR, DeBerardinis RJ, Mancuso A, Sayed N, Zhang XY, Pfeiffer HK, Nissim I, Daikhin E, Yudkoff M, McMahon SB, Thompson CB (2008). Myc regulates a transcriptional program that stimulates mitochondrial glutaminolysis and leads to glutamine addiction. Proc Natl Acad Sci U S A.

[45] Xiao H, Cheng S, Tong R, Lv Z, Ding C, Du C, Xie H, Zhou L, Wu J, Zheng S (2014). BAG3 regulates epithelial-mesenchymal transition and angiogenesis in human hepatocellular carcinoma. Lab Invest.

